# Fluctuations in External Peak Demands Across Quarters During Basketball Games

**DOI:** 10.3389/fphys.2022.868009

**Published:** 2022-04-12

**Authors:** Enrique Alonso Pérez-Chao, Miguel-Ángel Gómez, Pedro Lisboa, Juan Trapero, Sergio L. Jiménez, Alberto Lorenzo

**Affiliations:** ^1^ Facultad de Ciencias de la Actividad Física y del Deporte, Universidad Politécnica de Madrid, Madrid, Spain; ^2^ Department of Physical Activity and Sports Science, Universidad Alfonso X el Sabio, Villanueva de la Cañada, Spain; ^3^ Faculty of Sports Sciences, Universidad Europea de Madrid, Madrid, Spain; ^4^ Centre for Sport Studies, Universidad Rey Juan Carlos, Madrid, Spain

**Keywords:** most demanding scenarios, performance, sport, training, worst-case scenarios, team sport, game demands

## Abstract

The purpose of this study was to compare external peak demands (PDs) across quarters (Q) in basketball. Thirteen elite, junior, male basketball players were monitored using electronic performance tracking systems. There were studied intervals for different time windows to determine the external PD for distance (m); player load; distance covered in four different zones; accelerations; and decelerations. A mixed linear model was run to identify differences among quarters, and the auto-correlation function was carried out to determine fluctuations across the whole game. The results showed significant differences between Q1 vs. Q2 for distance, player load, and standing–walking distance; between Q1 vs. Q3 for distance, player load, and HSR; between Q1 vs. Q4 for distance, player load, standing–walking, and HSR; and between Q3 vs. Q4 for distance and player load. These findings suggest that external PD for running-based demands (distance, player load, and high-speed running) decrease across basketball games with the most notable declines occurring between the first and fourth quarters. Nevertheless, it is important to note that non-significant differences were found between quarters for several external PD variables (jogging, running, acceleration, and deceleration) across different time windows. Findings from the present study reinforce the importance of considering specific PD variables for different functions due to the specific insight each provides.

## Introduction

The main objectives during training sessions are to 1) prescribe the optimal training load (Aoki et al., 2017); 2) stimulate specific adaptations (Aoki et al., 2017; Foster et al., 2001); and 3) obtain the desired responses (Impellizzeri et al., 2018). In turn, quantifying the physical and physiological loads is important to understand the dose-response nature of the training process when establishing optimal training procedures (Sosa et al., 2021). One of the most common methods utilized by coaches during training sessions is exposing players to game demands. Then, there is a need to apply training strategies replicating competition performance demands (Alonso et al., 2020).

There are established technological tools, such as electronic performance tracking systems (EPTSs) including wearable microsensor technology (e.g., accelerometers or gyroscopes) (Chambers et al., 2015; Gabbett 2013) and local positional systems (LPSs) (Serpiello et al., 2018; Hodder et al., 2020) that provide reliable and valid measures about the physical game demands in indoor team sports. The use of this technology provides several advantages such as 1) to include the capability to monitor several players at once; 2) the time effectiveness of the analysis; and 3) the ability to receive real-time information (Aughey and Cameron, 2010).

The ability to sustain high-intensity accelerations, decelerations, and change of directions and landings, as well as the ability to cope with these peak demands, is essential for basketball success, due to the intermittent high-intensity nature of the sport (Montgomery et al., 2010; Stojanović et al., 2018). To date, most of the studies have investigated the match-play demands with a focus on the average values across entire games. However, understanding game demands using averages drastically underestimates the most demanding scenarios (MDS) of match-play. Therefore, this approach does not take the natural intermittence of the game into account and represents an incomplete procedure (Alonso et al., 2020).

In order to improve the research knowledge about the external game demands, the quantification of MDS (i.e., peak demands and worst-case scenario) experienced during games is essential to tailor unique training plans that better prepare players’ physical fitness while successfully executing key technical skills (Alonso et al., 2020). Then, analyzing external peak demands (PDs) may provide a different insight. The PD, defined as the most intense activity experienced by players for a selected variable across a specified timeframe of interest (Alonso et al., 2020; Alonso Perez-Chao et al., 2021), has been quantified for basketball players using many external load variables (e.g., player load and distance) and time windows (e.g., 1 min and 2 min) (Fox et al., 2020a; Alonso et al., 2020; Fox et al., 2020b; Vázquez-Guerrero et al., 2020; Vázquez-Guerrero and Garcia 2020; Alonso Perez-Chao et al., 2021). Additionally, moving average has been proved as the most accurate method to determine the peak intensities (Cunningham et al., 2018).

Concerning fluctuations, research findings carried out in basketball have revealed that average external physical demands decline across games, being higher during the first quarter than the last game quarter or overtime (Scanlan et al., 2015; García et al., 2020). According to external PD, a previous study suggested that peak intensities decrease across quarters (Fox et al., 2020b). Similarly, the literature related to other sports such as soccer showed higher external PD during the first half compared to the second (Oliva-Lozano et al., 2020; Bradley and Noakes 2013). These findings may be attributed to changes in tactical aspects or situational influences, including more stoppages and, consequently, a longer period of duration or accumulated fatigue (García et al., 2020; Bradley and Noakes 2013).

Existing basketball data related with most demanding scenarios in basketball have analyzed the impact of different factors on the PD encountered by players, including the effects of player position (Alonso et al., 2020; Fox et al., 2020a), score-line (Vázquez-Guerrero et al., 2020), congested-fixture (Pino-Ortega et al., 2019), age category (García et al., 2021), type of activity (Vázquez-Guerrero et al., 2020; García et al., 2022), phase of the season (Alonso et al., 2022), playing time (Alonso Perez-Chao et al., 2021), or accumulated playing time prior to external PD (Alonso Perez-Chao et al., 2021). However, only one previous study has examined the peak intensities encountered by players across game quarters in basketball, suggesting peak values decrease across match-play (Fox et al., 2020b). Nevertheless, this study was focused on an isolated parameter (player load). Consequently, more research should include a suite of physical load variables when quantifying PD to provide a more comprehensive understanding of player demands during basketball games.

Based on the limited understanding of the external peak fluctuations experienced by basketball players, there is a need to improve the research knowledge about fluctuations in peak intensities across quarters by analyzing different performance parameters (e.g., distance in different velocity thresholds, change of directions, accelerations, or decelerations). An understanding of these fluctuations in external peak requirements would allow basketball practitioners to develop more precise conditioning practices, optimizing the player’s performance across specific game periods, and develop strategies for greater precision when prescribing training and managing fatigue. Therefore, the aim of this study was to compare the external PD encountered by players across game quarters for different time windows (30 sec, 45 sec, 1 min, 2 min, and 5 min), using LPS and microtechnology. The hypothesis of the study suggests that external PD for most external physical parameters might decline among quarters across the game.

## Materials and Methods

### Sample

Elite, junior, male basketball players (*n* = 13, mean ± standard deviation: 16.62 ± 0.96 years, height 197.62 ± 8.01 cm, body mass 87.77 ± 7.74 kg) at international competitive level (Swann et al., 2015) were monitored during nine official home games. The sample size was estimated using the G*power 3.1 statistical software considering one single group with four repeated measures, an alpha level of 95%, a *p*-value lower than 0.5, and effect size values larger than 0.4. Then, a minimum sample of 12 participants was required. Exclusion criteria followed through the study was a minimum of 15 minutes of playing time on court per match, where all players from the team, that had less than 15 minutes of real time on court per match, were excluded from that activity, but not from the study. In this regard, players had to complete 15 min of playing time in at least five games for inclusion in the study. Furthermore, each player that did not invest a minimum of 5 minutes of playing time on the court per quarter was excluded from that quarter, but not from the game. Thus, quarter samples from each player were only retained in the final analysis if they completed a minimum of 15 min playing time in that particular game, 5 min playing time on court in that specific quarter, in at least five games.

Playing time is the time in minutes that each player invests on the court during the match, including when the ball is stopped (that is: free throws, when the referee is marking a fault). Rest periods between quarters or time-outs are not considered as playing time. Besides, data collected at the rest periods between quarters or time-outs, has not been taken into consideration and therefore has been excluded from the study. Overall, a total of *n* = 270 quarters (Q1 *n* = 70, Q2 *n* = 69, Q3 *n* = 69 and Q4 *n* = 62) played by the 13 players were included in analyses. The study was in accordance to the Declaration of Helsinki (Harriss and Atkinson 2014) and approved by the Institutional Review Board of the Polytechnic University of Madrid, Spain.

### Procedures

This descriptive study was carried out during the 2019–2020 season where game-play was conducted in line with official FIBA rules (i.e., 4 × 10 min quarters). During games, each player wore a monitoring device (ClearSky S7, Catapult Sports, Melbourne, Australia) inserted into a fitted neoprene vest under regular playing attire and positioned on the upper thoracic spine between the scapulae (Hodder et al., 2020). Each device contained microsensor technology consisting of an accelerometer (±16 g, 100 Hz), magnetometer (±4.900 µT, 100 Hz), and gyroscope (up to 2,000 deg/sec, 100 Hz). Each device was also interfaced with a LPS sampling at 10 Hz. The LPS was an ultra-wide band, 4 GHz transmitting system equipped with 24 anchors positioned around the perimeter of stadium. The LPS technology (ClearSky by Catapult) used in this study has been supported as valid in measuring distance, speed, and accelerations (Luteberget et al., 2018; Hodder et al., 2020; Serpiello et al., 2018), while similar LPS technology has been shown to be reliable (coefficient of variation (CV) <5%) in measuring distance and speed variables (Hoppe et al., 2018; Gómez-Carmona et al., 2019). All players were familiar with the monitoring technology as they had worn the devices during training sessions and games in the previous season. Devices were turned on ∼20–40 min before the warm-up phase prior to each game, and players wore the same device throughout the study period to avoid inter-unit variation in outputs (Castellano et al., 2011; Johnston et al., 2014; Nicolella et al., 2018).

### Variables

Different data-filtering methods can substantially affect the number of high-intensity movements detected using LPS devices (Varley et al., 2017). That is the reason practitioners should determine a criterion and should be consistent with their choice (Varley et al., 2017; Malone et al., 2017). Furthermore, researchers should include detailed information in practical reports and research publications (Varley et al., 2017). Dwell time is the minimum effort duration (MED) that should be maintained to be counted. Some studies have shown that changes in the MED as small as 0.1 s substantially modify the results (Varley et al., 2017). The following acceleration/deceleration dwell time was chosen for this study (dwell time: 0.3 s) given values between 0.3 and 0.4 has been specified as the most common to consider in basketball (Svilar and Jukic. 2018; Alonso et al., 2020; Salazar et al., 2020).

To determine the external PD, first, we extracted the raw data in each 1 s interval for each player. After that, we exported the data to a custom-built Microsoft Excel spreadsheet (version 16.0, Microsoft Corporation, Redmond, WA) for further analysis; lastly, we analyzed different intervals using rolling sum. This procedure is more accurate when determining the most intense periods than the FIXED method (Cunningham et al., 2018) and has been previously used in several studies (Cunningham et al., 2018; Fox et al., 2020a; Alonso et al., 2020; Fox et al., 2020b; Vázquez-Guerrero et al., 2020; Alonso Perez-Chao et al., 2021). Rolling averages were stopped at the end of each quarter. Thus, rolling started at the beginning of each quarter and stopped at the end of the same quarter. The PD, for each player, parameter, and quarter, was determined for five different time windows (30 and 45 s and 1, 2, and 5 min). These time windows were chosen given they have been identified as the most practical to consider in basketball (Alonso et al., 2020; Fox et al., 2020b; Vázquez-Guerrero et al., 2020; Alonso Perez-Chao et al., 2021). In this regard, the rationale behind the selection of the short windows (i.e., 30, 45 s, 1 min) was based on durations longer than 3 min are very unlikely (Zhang et al., 2019) while the selection of large window (i.e., 5 min) provide valuable insight given this duration is commonly implemented when prescribing various drills during training scenarios (Alonso Perez-Chao et al., 2021). Thus, allows basketball practitioners to compare PD with the game-based drills during practices, since, although it depends on the staff routines, the game-based drills duration usually is ranged between 4–10 min (O’Grady et al., 2021).

The parameters recorded were distance (m); player load; distance covered in four different zones: zone 1: standing–walking, zone 2: jogging, zone 3: running, zone 4: high speed running (HSR), accelerations (>2 ms^−2^, dwell time = 0.3 s) and decelerations (>2 ms^−2^, dwell time = 0.3 s). Total distance (TD) is meters covered by the players while on the field. Player load™ (PL) considers the instantaneous rate of change of acceleration in three different planes (x-, y-, and z-axis) measured in arbitrary units (au) (Brown and Greig 2015). This parameter has been used in several basketball studies (Reina et al., 2019; Fox et al., 2020a; Alonso et al., 2020) and the formula is √ (ay1–ay-1) 2 + √ (ax1–ax-1) 2 + √ (az1–az-1)2/100 (Brown and Greig 2015), where fwd indicates movement in the anterior-posterior direction, side indicates movement in the medial-lateral direction, up indicates vertical movement, and t represents time. The speed zones selected were classified into the following four absolute velocity thresholds (Sosa et al., 2021) (Table 1).

**TABLE 1:** Absolute velocity thresholds.

Zones	Speed
Zone 1 (standing–walking)	<7 km·h^−1^
Zone 2 (jogging)	7.01–14 km·h^−1^
Zone 3 (running)	14.01–18 km·h^−1^
Zone 4 (high-speed running)	>18.01 km·h^−1^

### Statistical Analysis

Mean ± standard deviation (SD) and the coefficient of variation (CV%) were calculated for each external physical variable. The Mixed Linear Model (MLM) for repeated measures was carried out to identify differences among quarters across the game. The adjustment for multiple comparisons was performed using Bonferroni and the mean difference is considered significant at *p* < 0.05. Effect sizes (ES) for all pairwise comparisons were defined, as follows: ≤0.2, trivial; >0.2, small; >0.6, moderate; >1.2, large; >2.0, very large; and >4.0, nearly perfect (Hopkins et al., 2009). The auto-correlation function (ACF) was run to determine external peak fluctuation across the whole game for each parameter, is game, and player. Descriptive analysis, MLMs, post-hoc tests, and ACFs were conducted using the statistical software IBM SPSS for Windows (Version 23, IBM Corp) while ES were calculated using a customized Microsoft Excel spreadsheet (version 16.0, Microsoft Corporation, Redmond, WA).

## Results

**FIGURE 1:**
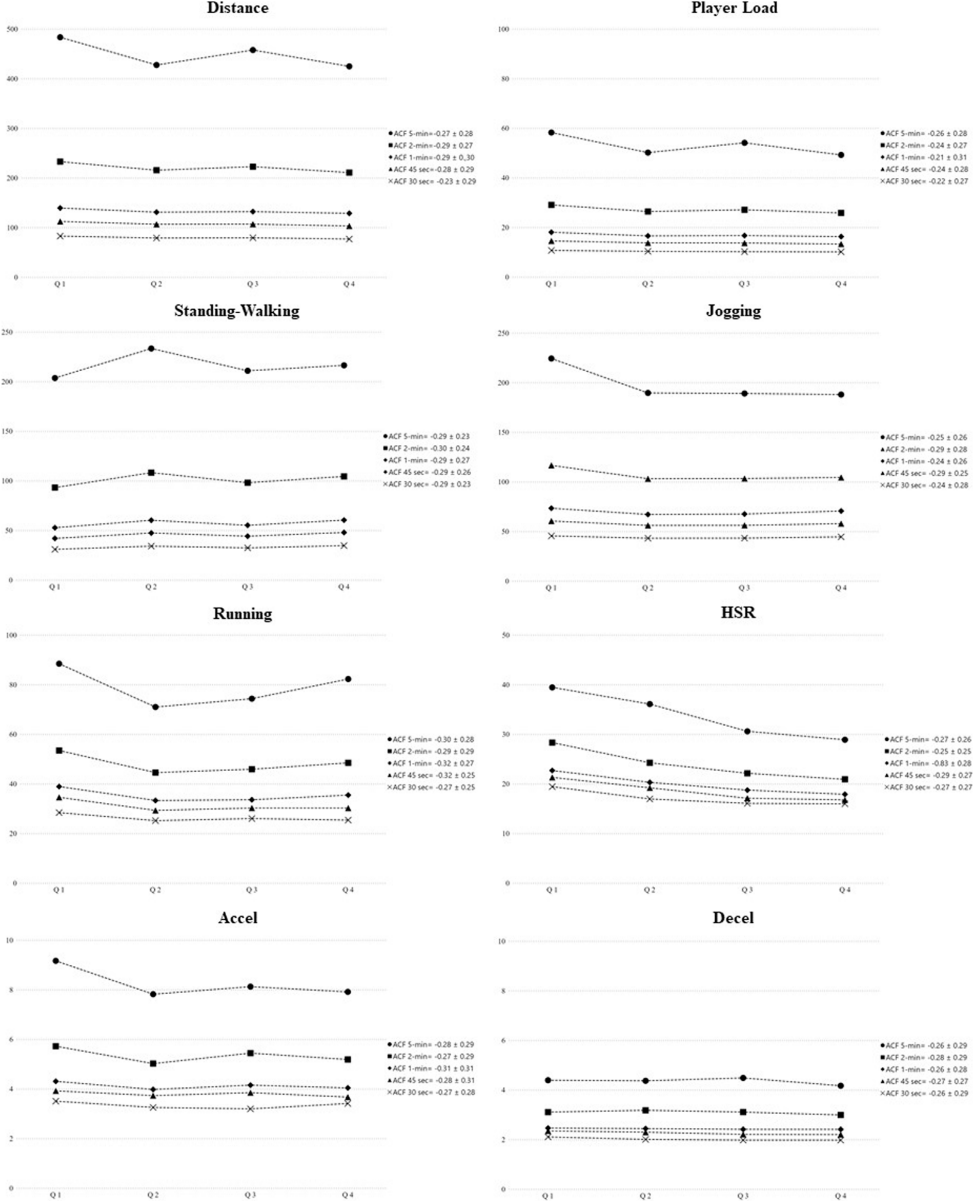
Fluctuations across quarters for each parameter and time window.

Descriptive analysis per quarter (mean ± SD and CV %) and fluctuations across game quarters for each sample duration are presented in Table 2 and Figure 1, respectively. For the 30, 45 sec, 1 min, 2 min, and 5 min sample durations, differences in jogging, running, acceleration, and deceleration between quarters were non-significant. However, there were significant differences across the game in distance, player load, standing–walking, or HSR variables. Pairwise comparisons between quarters for each variable and time window are shown in Table 3.

**TABLE 2:** Descriptive analysis per quarter for different time windows.

Window	Parameter	Q1 (*n* = 70)		Q2 (*n* = 69)		Q3 (*n* = 69)		Q4 (*n* = 62)	
		Mean ± SD	CV (%)	Mean ± SD	CV (%)	Mean ± SD	CV (%)	Mean ± SD	CV (%)
30 s	Distance	83.16 ± 8.65	10	79.38 ± 9.05	11	79.72 ± 7.66	10	77.23 ± 8.27	11
	Player load	10.80 ± 1.50	14	10.44 ± 1.67	16	10.31 ± 1.37	13	10.22 ± 1.67	16
	Standing–walking	31.05 ± 5.06	16	34.31 ± 7.31	21	32.55 ± 4.87	15	34.84 ± 6.44	18
	Jogging	45.67 ± 10.49	22	43.35 ± 10.03	23	43.44 ± 7.70	18	44.71 ± 9.94	22
	Running	28.47 ± 7.22	25	25.25 ± 6.92	27	26.07 ± 5.61	22	25.46 ± 14.11	55
	HSR	19.46 ± 9.29	47	19.99 ± 7.70	45	16.10 ± 8.07	50	16.01 ± 7.82	49
	Accel	3.51 ± 1.28	36	3.26 ± 1.20	36	3.20 ± 1.21	38	3.42 ± 1.43	42
	Decel	2.11 ± 0.94	44	2.01 ± 0.83	41	1.99 ± 0.98	49	1.98 ± 0.84	42
45 s	Distance	112.76 ± 11.74	10	107.05 ± 12.30	11	107.24 ± 10.23	10	103.52 ± 11.44	11
	Player load	14.63 ± 1.96	13	13.84 ± 2.34	16	13.79 ± 1.83	13	13.38 ± 2.25	17
	Standing–walking	42.11 ± 7.74	18	47.53 ± 12.16	25	44.32 ± 7.83	18	48.06 ± 10.80	22
	Jogging	60.71 ± 17.22	28	56.29 ± 15.33	27	56.35 ± 10.03	18	58.07 ± 15.03	26
	Running	34.64 ± 9.35	26	29.37 ± 8.22	28	30.33 ± 7.73	25	21.29 ± 7.73	70
	HSR	21.34 ± 10.51	49	19.24 ± 9.66	50	17.13 ± 9.13	53	16.80 ± 8.16	49
	Accel	3.93 ± 1.43	36	3.74 ± 1.32	35	3.86 ± 1.48	38	3.68 ± 1.61	44
	Decel	2.36 ± 1.02	43	2.30 ± 1.05	45	2.22 ± 1.07	48	2.21 ± 0.93	42
1 min	Distance	139.77 ± 14.17	10	131.36 ± 15.64	11	132.47 ± 12.58	9	129.05 ± 14.03	11
	Player load	18.13 ± 2.37	13	16.65 ± 2.90	17	16.78 ± 2.41	14	16.37 ± 2.84	17
	Standing–walking	52.86 ± 10.43	19	60.40 ± 17.16	28	55.34 ± 11.08	20	60.55 ± 15.64	26
	Jogging	75.57 ± 23.13	31	67.29 ± 21.05	31	67.72 ± 13.00	19	70.78 ± 20.61	29
	Running	38.98 ± 11.14	28	33.36 ± 9.33	28	33.66 ± 8.95	27	35.53 ± 28.58	80
	HSR	22.72 ± 11.19	49	20.34 ± 10.31	50	18.77 ± 10.22	54	17.92 ± 10.22	50
	Accel	4.31 ± 1.49	34	3.99 ± 1.44	36	4.16 ± 1.59	38	4.05 ± 1.78	44
	Decel	2.47 ± 1.09	43	2.45 ± 1.08	44	2.42 ± 1.16	48	2.42 ± 1.03	43
2 min	Distance	233.12 ± 27.90	11	215.94 ± 27.02	12	223.00 ± 23.46	11	211.16 ± 25.15	12
	Player load	29.15 ± 4.37	15	26.50 ± 4.64	17	27.17 ± 4.32	16	25.91 ± 4.29	17
	Standing–walking	93.32 ± 22.79	24	108.40 ± 34.28	31	98.23 ± 23.28	24	104.56 ± 25.69	25
	Jogging	116.79 ± 51.07	43	103.33 ± 40.45	19	103.55 ± 21.03	20	104.57 ± 42.73	41
	Running	52.52 ± 16.43	30	44.60 ± 12.72	28	45.97 ± 13.25	29	48.54 ± 58.12	120
	HSR	28.35 ± 13.69	48	24.29 ± 12.55	51	22.17 ± 12.28	55	20.95 ± 11.04	53
	Accel	5.73 ± 1.95	34	5.03 ± 1.91	38	5.45 ± 2.08	38	5.19 ± 2.35	45
	Decel	3.11 ± 1.44	46	3.19 ± 1.42	44	3.12 ± 1.45	47	3.00 ± 1.28	43
5 min	Distance	483.74 ± 69.42	14	427.8 ± 61.78	14	458.01 ± 64.39	14	425.08 ± 64.09	15
	Player load	58.34 ± 10.62	18	50.23 ± 10.61	21	54.19 ± 9.60	18	49.31 ± 10.24	21
	Standing–walking	203.74 ± 58.91	28	233.53 ± 91.49	39	211.07 ± 56.99	27	216.56 ± 55.95	26
	Jogging	224.50 ± 132.35	58	189.78 ± 100.37	52	189.24 ± 46.16	24	188.16 ± 106.97	57
	Running	88.52 ± 31.11	35	71.05 ± 21.09	29	74.41 ± 21.84	29	82.34 ± 140.33	170
	HSR	39.48 ± 20.50	51	36.12 ± 20.78	57	30.63 ± 18.47	60	28.93 ± 18.13	63
	Accel	9.17 ± 3.51	38	7.83 ± 3.42	43	8.13 ± 2.98	37	7.92 ± 3.66	46
	Decel	4.40 ± 2.01	45	4.38 ± 1.96	44	4.49 ± 2.34	52	4.18 ± 2.32	55

**TABLE 3:** Pairwise comparisons between quarters for each parameter and time window.

Parameter	Window	F	*p*	Pairwise comparisons											
				Q1vsQ2		Q1vsQ3		Q1vsQ4		Q2vsQ3		Q2vsQ4		Q3vsQ4	
				*p*	ES	*p*	ES	*p*	ES	*p*	ES	*p*	ES	*p*	ES
Distance	30 s	5.76	0.001	*p* = 0.049*	0.43	*p* = 0.095	0.42	*p* < 0.000*	0.7	*p* = 1.000	−0.04	*p* = 0.850	0.25	*p* = 0.536	0.31
	45 s	7.513	0.000	*p* = 0.020*	0.47	*p* = 0.027*	0.5	*p* < 0.000*	0.8	*p* = 1.000	−0.02	*p* = 0.467	0.3	*p* = 0.378	0.34
	1 min	7.361	0.000	*p* < 0.003*	0.56	*p* = 0.014*	0.54	*p* < 0.000*	0.76	*p* = 1.000	−0.08	*p* = 0.990	0.16	*p* = 0.990	0.26
	2 min	9.191	0.000	*p* < 0.001*	0.63	*p* = 0.128	0.39	*p* < 0.000*	0.82	*p* = 0.654	−0.28	*p* = 1.000	0.18	*p* = 0.055	0.49
	5 min	12.51	0.000	*p* < 0.000*	0.85	*p* = 0.117	0.38	*p* < 0.000*	0.88	*p* = 0.039	−0.48	*p* = 1.000	0.04	*p* = 0.023*	0.51
Player load	30 s	1.85	0.138	*p* = 1.000	0.23	*p* = 0.356	0.34	*p* = 0.197	0.37	*p* = 1.000	0.09	*p* = 1.000	0.13	*p* = 1.000	0.06
	45 s	4.184	0.006	*p* = 0.165	0.37	*p* = 0.112	0.44	*p* < 0.004*	0.59	*p* = 1.000	0.02	*p* = 1.000	0.2	*p* = 1.000	0.2
	1 min	6.101	0.000	*p* < 0.006*	0.56	*p* = 0.016*	0.56	*p* < 0.001*	0.68	*p* = 1.000	−0.05	*p* = 1.000	0.1	*p* = 1.000	0.16
	2 min	6.991	0.000	*p* < 0.003*	0.59	*p* = 0.049*	0.46	*p* < 0.000*	0.75	*p* = 1.000	−0.15	*p* = 1.000	0.13	*p* = 0.619	0.29
	5 min	11.07	0.000	*p* < 0.000*	0.76	*p* = 0.103	0.41	*p* < 0.000*	0.86	*p* = 0.142	−0.39	*p* = 1.000	0.09	*p* = 0.040*	0.49
Standing–walking	30 s	5.651	0.001	*p* < 0.009	−0.52	*p* = 0.834	−0.30	*p* < 0.002*	−0.66	*p* = 0.509	0.28	*p* = 1.000	−0.08	*p* = 0.175	−0.40
	45 s	5.619	0.001	*p* < 0.007*	−0.53	*p* = 1.000	−0.28	*p* < 0.003*	−0.64	*p* = 0.321	0.31	*p* = 1.000	−0.05	*p* = 0.172	−0.40
	1 min	5.251	0.002	*p* < 0.008	−0.53	*p* = 1.000	−0.23	*p* < 0.009*	−0.59	*p* = 0.186	0.35	*p* = 1.000	−0.01	*p* = 0.184	−0.39
	2 min	4.301	0.060	*p* < 0.006*	−0.31	*p* = 1.000	0.05	*p* = 0.099	−0.22	*p* = 0.158	0.35	*p* = 1.000	0.13	*p* = 1.000	−0.26
	5 min	2.464	0.063	*p* = 0.057	−0.39	*p* = 1.000	−0.13	*p* = 1.000	−0.22	*p* = 0.304	0.29	*p* = 0.902	0.22	*p* = 1.000	−0.10
Jogging	30 s	0.935	0.424	*p* = 0.917	0.23	*p* = 1.000	0.35	*p* = 1.000	0.09	*p* = 1.000	0.10	*p* = 1.000	−0.14	*p* = 1.000	−0.26
	45 s	1.412	0.239	*p* = 0.448	0.27	*p* = 0.472	0.31	*p* = 1.000	0.16	*p* = 1.000	0.00	*p* = 1.000	−0.12	*p* = 1.000	−0.14
	1 min	1.537	0.205	*p* = 0.367	0.37	*p* = 0.485	0.42	*p* = 1.000	0.22	*p* = 1.000	−0.02	*p* = 1.000	−0.17	*p* = 1.000	−0.18
	2 min	1.835	0.141	*p* = 0.291	0.29	*p* = 0.314	0.34	*p* = 0.487	0.26	*p* = 1.000	−0.01	*p* = 1.000	−0.03	*p* = 1.000	−0.03
	5 min	2.141	0.905	*p* = 0.258	0.30	*p* = 0.240	0.35	*p* = 0.237	0.30	*p* = 1.000	0.01	*p* = 1.000	0.02	*p* = 1.000	0.01
Running	30 s	1.924	0.126	*p* = 0.197	0.46	*p* = 0.671	0.37	*p* = 0.315	0.27	*p* = 1.000	−0.13	*p* = 1.000	−0.02	*p* = 1.000	0.06
	45 s	2.463	0.063	*p* = 0.083	0.60	*p* = 0.260	0.50	*p* = 0.295	1.55	*p* = 1.000	−0.12	*p* = 1.000	1.01	*p* = 1.000	1.17
	1 min	1.795	0.148	*p* = 0.243	0.55	*p* = 0.314	0.53	*p* = 1.000	0.16	*p* = 1.000	−0.03	*p* = 1.000	−0.10	*p* = 1.000	−0.09
	2 min	1.171	0.321	*p* = 0.504	0.54	*p* = 0.859	0.44	*p* = 1.000	0.10	*p* = 1.000	−0.11	*p* = 1.000	−0.10	*p* = 1.000	−0.06
	5 min	0.869	0.458	*p* = 0.861	0.66	*p* = 1.000	0.52	*p* = 1.000	0.06	*p* = 1.000	−0.16	*p* = 1.000	−0.12	*p* = 1.000	−0.08
HSR	30 s	2.631	0.050	*p* = 0.460	-0.06	*p* = 0.099	0.39	*p* = 0.100	0.40	*p* = 1.000	0.49	*p* = 1.000	0.51	*p* = 1.000	0.01
	45 s	3.412	0.018	*p* = 1.000	0.21	*p* = 0.052	0.43	*p* = 0.036*	0.48	*p* = 1.000	0.22	*p* = 0.834	0.27	*p* = 1.000	0.04
	1 min	2.907	0.035	*p* = 1.000	0.22	*p* = 0.135	0.37	*p* = 0.043*	0.45	*p* = 1.000	0.15	*p* = 1.000	0.24	*p* = 1.000	0.08
	2 min	4.661	0.003	*p* = 0.322	0.31	*p* = 0.021*	0.48	*p* < 0.004*	0.59	*p* = 1.000	0.17	*p* = 0.746	0.28	*p* = 1.000	0.10
	5 min	4.245	0.006	*p* = 1.000	0.11	*p* = 0.046*	0.40	*p* = 0.012*	2.01	*p* = 0.585	0.28	*p* = 0.210	1.84	*p* = 1.000	1.66
Accel	30 s	0.875	0.455	*p* = 1.000	0.20	*p* = 0.896	0.25	*p* = 1.000	0.07	*p* = 1.000	0.05	*p* = 1.000	−0.12	*p* = 1.000	−0.17
	45 s	0.405	0.749	*p* = 1.000	0.14	*p* = 1.000	0.05	*p* = 1.000	0.16	*p* = 1.000	−0.09	*p* = 1.000	0.04	*p* = 1.000	0.12
	1 min	0.587	0.624	*p* = 1.000	0.22	*p* = 1.000	0.10	*p* = 1.000	0.16	*p* = 1.000	−0.11	*p* = 1.000	−0.04	*p* = 1.000	0.07
	2 min	1.525	0.208	*p* = 0.275	0.36	*p* = 1.000	0.14	*p* = 0.820	0.25	*p* = 1.000	−0.21	*p* = 1.000	−0.08	*p* = 1.000	0.12
	5 min	2.332	0.075	*p* = 0.116	0.39	*p* = 0.418	0.32	*p* = 0.204	0.35	*p* = 1.000	−0.09	*p* = 1.000	−0.03	*p* = 1.000	0.06
Decel	30 s	0.324	0.808	*p* = 1.000	0.11	*p* = 1.000	0.12	*p* = 1.000	0.15	*p* = 1.000	0.02	*p* = 1.000	0.04	*p* = 1.000	0.01
	45 s	0.333	0.801	*p* = 1.000	0.06	*p* = 1.000	0.13	*p* = 1.000	0.15	*p* = 1.000	0.08	*p* = 1.000	0.09	*p* = 1.000	0.01
	1 min	0.036	0.991	*p* = 1.000	0.02	*p* = 1.000	0.04	*p* = 1.000	0.05	*p* = 1.000	0.03	*p* = 1.000	0.03	*p* = 1.000	0.00
	2 min	0.203	0.895	*p* = 1.000	−0.06	*p* = 1.000	−0.01	*p* = 1.000	0.08	*p* = 1.000	0.05	*p* = 1.000	0.14	*p* = 1.000	0.09
	5 min	0.247	0.863	*p* = 1.000	0.01	*p* = 1.000	−0.04	*p* = 1.000	0.10	*p* = 1.000	−0.05	*p* = 1.000	0.09	*p* = 1.000	0.13

The mean difference is considered significant at *p* < 0.05 (*).

For Distance, there was a significant decline for all sample durations between Q1 vs. Q2 (30 s: small ES = 0.43; 45 s: small ES = 0.47; 1 min: small ES = 0.56; 2 min: moderate ES = 0.63; 5 min: moderate ES = 0.85), and Q1 vs. Q4 (30 s: moderate ES = 0.70; 45 s: moderate ES = 0.80; 1 min: moderate ES = 0.76; 2 min: moderate ES = 0.82; 5 min: moderate ES = 0.88). Besides, significant differences were found between Q1 vs. Q3 (45 s: small ES = 0.50; 1 min: small ES = 0.54) and Q3 vs. Q4 (5 min: small ES = 0.51). Concerning player load, differences were found among Q1 vs. Q2 (1 min: small ES = 0.56; 2 min: small ES = 0.59; 5 min: moderate ES = 0.76), Q1 vs. Q3 (1 min: small ES = 0.56; 2 min: small ES = 0.43), Q1 vs. Q4 (45 s: small ES = 0.59; 1 min: moderate ES = 0.68; 2 min: moderate ES = 0.75; 5 min: moderate ES = 0.86) and Q3 vs. Q4 (5 min: small ES = 0.49). Moreover, differences were found between Q1 vs. Q2 (45 s: small ES = −0.53; 2 min: small ES = −0.31) and Q1 vs. Q4 (30 s: moderate ES = −0.66; 45 s: moderate ES = −0.64; 1 min: small ES = −0.59) for standing–walking variable. For HSR, differences were found between Q1 vs. Q3 (2 min: small ES = 0.48; 5 min: small ES = 0.40), and Q1 vs. Q4 (45 s: small ES = 0.48; 1 min: small ES = 0.45; 2 min: small ES = 0.59; 5 min: very large ES = 2.01). Additionally, ACF revealed a tendency where external PD decline across quarters (Figure 1).

## Discussion

The purpose of the present study was to compare the external PD encountered by players across game quarters considering different time windows (30 s, 45 s, 1 min, 2 min, and 5 min), using LPS and microtechnology. There were several novel findings from the data analyzed that can help to achieve a better understanding of external physical PD during games: significant decreases across the whole game with the most notable declines in external peak values occurring between the first and fourth quarters for total distance, player load, standing–walking, and high-speed running. However, differences between quarters in jogging, running, acceleration, and deceleration, for all sample durations, were non-significant.

Since TD and PL are correlated (Heishman et al., 2020), the results in both variables were similar, which means that there are significant differences in most sample durations between Q1 vs. Q2 and Q1 vs. Q4 as was previously identified (Fox et al., 2020b). For HSR, significant differences between Q1 vs. Q4 were found (45 s; 1 min; 2 min; and 5 min). In addition, based on ACF analysis, the current data revealed a tendency where, despite high levels of variability, external physical PD declines across games. These findings suggest that PD over 45 s related with physiological running-based demands decrease across games. Furthermore, there were differences in standing–walking distance between Q1 vs. Q2 (45 s; 2 min) and Q1 vs. Q4 (30 s; 45 s; and 1 min). Thus, standing–walking was the only external physical variable that showed higher peak values in the last quarter compared to the beginning of the game. In this regard, the increase in standing–walking variable during the last quarter may be associated with situation-related variables such as game pace, the call of more time-outs, or stoppages in play due to free-throws from bonus situations.

There are many factors contributing to the decline in TD, PL, and HSR, mainly in larger sample duration. These findings may be associated with fatigue mechanisms reducing the ability to produce a given force or power output (Gibson and Noakes 2004; Green 1997). For instance, glycogen depletion, muscle damage, action potential interruption, and excitation–contraction coupling failure (Noakes et al., 2005; Gibson and Noakes 2004; Green 1997) resulting from basketball activity may impede the ability of players to sustain high-intensity activity outputs during the last passages of the game. In this regard, a recent study carried out in basketball showed players who undertake less playing time overall and prior to each PD episode can reach higher peak external for total distance (30 s to 2 min windows), PL (1 min to 2 min windows), and HSR distance (30 s and 5 min windows) than players who participated more playing time overall and before each external PD episode during games. These outcomes suggest players cannot attain as high peak external demands when accumulating more playing time leading into intense passages of player during games (Alonso Perez-Chao et al., 2021). In addition to fatigue mechanisms, the decline in TD, PL, and HSR may be related to situational variables such as competition type or opponent’s strength (Oliva-Lozano et al., 2020; Vázquez-Guerrero et al., 2020), score-line (Vázquez-Guerrero et al., 2020), congestion schedule (Edwards et al., 2018), next match location (Oliveira et al., 2021), player position/role (Alonso et al., 2020; Rojas-Valverde et al., 2020; Vázquez-Guerrero et al., 2020; Díez et al., 2021), the inherent demands of the specific player characteristics, time played, tactical strategies, pace or more time-outs, and stoppages in play for a change in possession of free-throw during the last quarter.

Findings from the present study also showed, most differences for all external physical variables and time windows were found between Q1 vs. Q2 and Q1 vs. Q4. A recent study that analyzed the differences in PD for PL between quarters in basketball reached similar conclusions, being higher external PD during the first quarter (Fox et al., 2020b). These findings may be related to the break between halves allowing a better recovery opportunity than between quarters. Similar results were found in soccer, where PD was higher during the first half than the second half (Casamichana et al., 2019; Oliva-Lozano et al., 2020). These results could provide an explanation for the lack of any clear differences between the Q2 vs. Q3, suggesting that the break time has an important role in fatigue recovery. Another point to consider is the fact that, as the sample duration becomes larger, greater differences arise between the first and second half. Nevertheless, future studies may confirm if this phenomenon (lowest PD during the last quarters) is due to fatigue or as a consequence of those previous factors mentioned (contextual factors). We should therefore clarify the reasons why external PD is higher during Q1 than Q4 and more specific research is necessary to study its effect on PD.

Our findings can offer useful practical application in many ways for basketball practitioners during practices with unselected or fringe players and rehabilitation sessions (Alonso et al., 2020). For example, in order to allow unselected players or athletes who only play “junk minutes” to replicate external peak requirements to balance their workload (Alonso et al., 2020) the values acquired during Q1 (or Q4 in the case of standing–walking) should be used as reference values instead of the peak intensities shown during the rest of the periods. These outcomes would also provide useful evidence for coaching staff when preparing specific game-based drills, tapering, or during the return to train/play/performance rehabilitation process.

Some limitations should be considered when interpreting the current findings. First, external PD in 5 min window could be conditioned by time outs that were not excluded. In this regard, variables such as score-line, playing position, player’s role, the time before each PD, quality of opposition, tactical aspects, or other factors that could have directly or indirectly influenced the study results were not controlled for. Accordingly, as a way to improve the research knowledge of peak fluctuations, these previous factors mentioned should be investigated in further research. Second, another limitation of the current study is the use of small sample size (a single elite, junior, male basketball team with 13 players). Specifically, it cannot be assumed that the external PD differences observed between quarters are generalized to other samples (e.g., female players or professional players). In this regard, further research should include different competitions and analyze contextual factors such as position differences or away games.

## Conclusion

This study provides useful information for coaching staff on the external PD, considering peak intensities for running-based demands (distance, player load, and high-speed running) decrease across basketball games with the most notable declines occurring between the first and fourth quarters. Nevertheless, it is important to note that non-significant differences were found between quarters for several external PD variables (jogging, running, acceleration, and deceleration) across different time windows. Findings from the present study reinforce the importance of considering specific PD variables for different functions due to the specific insight each provides.

## Data Availability

The original contributions presented in the study are included in the article/Supplementary Material, further inquiries can be directed to the corresponding author.

## Abbreviations

ACF, Auto-correlation function; HSR, High-speed running; LMM, Linear mixed model; LPS, Local positional system; MED, Minimum effort duration; PD, Peak demand; PL, Player load; Q, Quarter; TD, Total distance.
